# Ultraviolet irradiation improves the hydrophilicity and osteo-conduction of hydroxyapatite

**DOI:** 10.1186/s13018-020-01949-3

**Published:** 2020-09-18

**Authors:** Sho Kaneko, Yuji Yamamoto, Kanichiro Wada, Gentaro Kumagai, Yoshifumi Harada, Ryota Yamauchi, Yasuyuki Ishibashi

**Affiliations:** Department of Orthopaedic Surgery, Hirosaki University Graduate School of Medicine, 5 Zaifu-cho, Hirosaki, Aomori, 036-8562 Japan

**Keywords:** Ultraviolet, Hydroxyapatite, Osseointegration, Photofunctionalisation

## Abstract

**Background:**

Treating a titanium or titanium alloy implant with ultraviolet (UV) light is known to improve its associated cell growth and osseointegration. However, little is known about the effect of UV irradiation on hydroxyapatite (HA), which is also used frequently in orthopaedic and dental surgery. Here we examined the effect of UV irradiation on the hydrophilicity of HA, and on its osteoconduction ability in rats.

**Methods:**

HA implants of low and high porosity were treated with UV light, and photofunctionalisation was assessed by the contact angle of a water drop on the surface. HA implants were also inserted into rat femurs, and the rats were killed 2 or 4 weeks later. The bone volume and bone area ratio were calculated from microcomputed tomography and histological data.

**Results:**

The contact angle of a water drop on HA implants of both porosities was significantly reduced after UV irradiation. In the rat femurs, there was no significant difference in the bone volume between the UV light-treated and control implants at 2 or 4 weeks. The bone area ratio for the UV light-treated versus control implants was significantly increased at 2 weeks, but there was no significant difference at 4 weeks.

**Conclusions:**

The surface of UV-irradiated HA disks was hydrophilic, in contrast to that of non-irradiated HA disks. Photofunctionalisation accelerated the increase in the bone area ratio in the early healing stage. This technology can be applied to surgical cases requiring the early fusion of bone and HA.

## Background

It was recently shown that irradiating implants with ultraviolet (UV) light improves their associated cell growth and bone binding ability (osseointegration) [1, 2]. Irradiating the surface of titanium (Ti) or titanium alloy (Ti6Al4V) with ultraviolet rays of a short wavelength improves the material’s osseointegration capacity. Irradiation changes the surface structure of Ti such that radicals are excited and the hydrophilicity is increased. Yamauchi et al. reported that the bone-implant contact (BIC) ratio for both Ti and Ti6Al4V UV-treated implants significantly increased at 2 weeks [3]. UV irradiation was also found to have an antimicrobial effect in the early stage after implantation [4].

Hydroxyapatite [Ca10(PO4)6(OH)2] (HA) is commonly used in orthopaedic and dental surgery because of its osteoconductivity and good biocompatibility. HA is used frequently in orthopaedic surgery, for example, to fill in bone defects, or to coat artificial joints, which promotes early fixation. To treat cervical myelopathy, HA is used in spinous process-splitting laminoplasty. In this procedure, the spines are split sagittally, and trapezoid-shaped HA spacers are inserted between the two halves to maintain an enlarged spinal canal [5]. Although it was reported that porous HA was a better material for osteoinduction [6], cases were reported in which bone fusion is insufficient or in which complications like dislocation occur, which were related to HA’s osteogenesis ability [7].

Previous reports showed that UV irradiation physicochemically alters the HA surface [8–10]. Hydroxyl radicals on the surface of irradiated HA can increase its wettability and hydrophilicity, similar to the effect of photofunctionalised Ti. In an in vitro study using HA/poly-l-lactic acid (PLLA), UV treatment improved the surface hydrophilicity without changing the mechanical strength, and cell adhesion to UV-treated HA/PLLA was significantly improved [11]. Therefore, the cell-adhesion ability to HA may increase by UV treatment due to the change in the surface wettability and hydrophilicity, leading to the improved osteoconductive ability of HA.

Here we examined the effect of UV irradiation on the hydrophilicity of HA and on HA’s osteoconduction ability in rats. We hypothesised that UV irradiation would increase the hydrophilicity of the HA surface layer as it does for Ti. To date, there has been little research on the influence of UV irradiation on HA in live animals.

## Materials and methods

We performed an in vitro study to demonstrate the effect of UV irradiation on the hydrophilicity of the HA surface and an in vivo study to demonstrate the effect of UV irradiation on the osteoconductive ability of HA. In the in vivo study, HA implants were inserted into rat femurs, and radiological analyses using microcomputed tomography and histological analyses using undecalcified specimens were performed. The study protocol (ethical code number: M16018) was approved by the Animal Research Committee of Hirosaki University, and all experiments were performed in accordance with the Rules for Animal Experimentation of Hirosaki University.

### Hydrophilicity of the hydroxyapatite surface

**Fig. 1:**
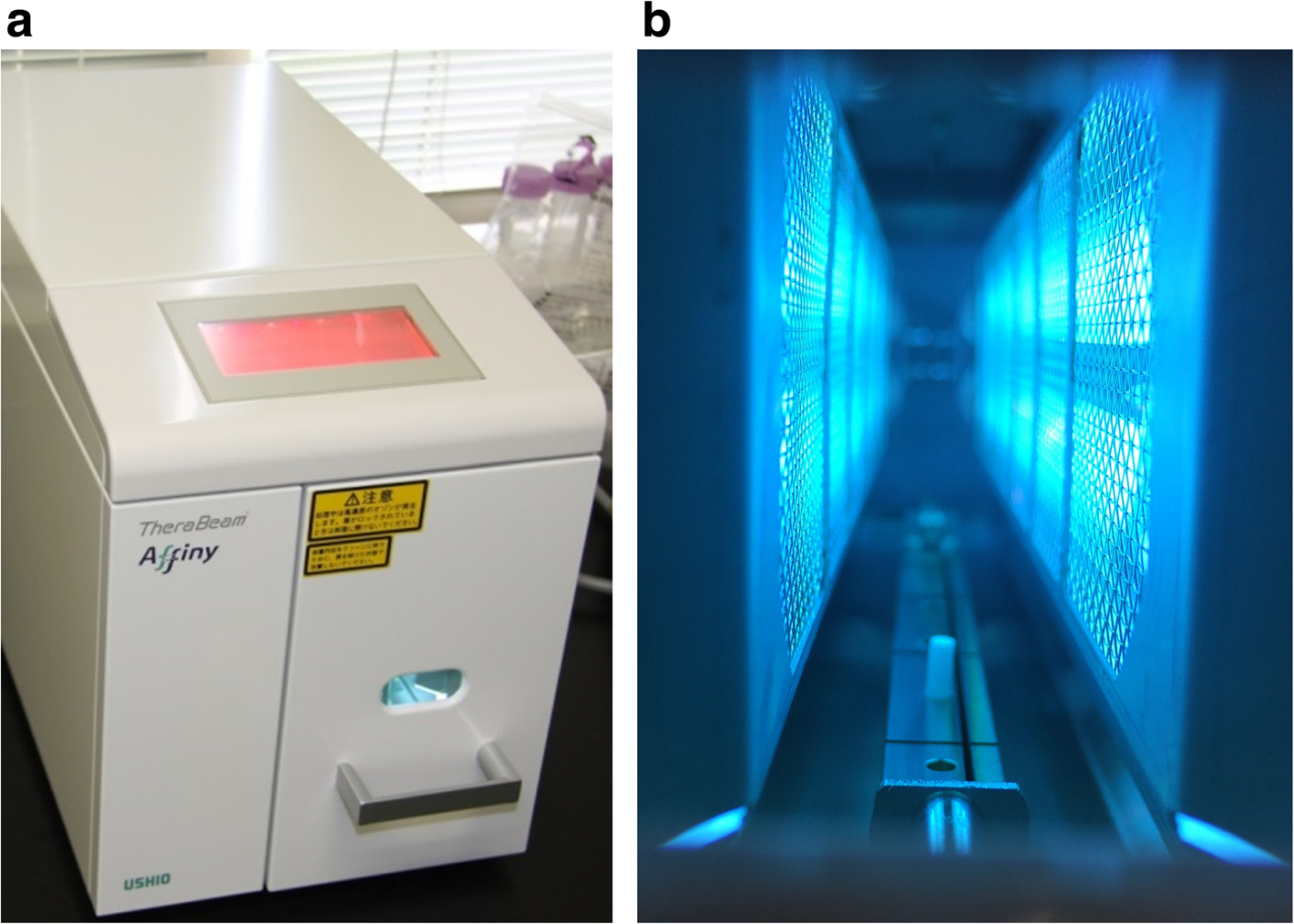
Irradiation of HA implants with UV light. **a** UV photo device (TheraBeam® Affinity, Ushio Inc., Tokyo, Japan). **b** Implants were subjected to UV irradiation for 15 min

HA disks (diameter 15 mm, height 3 mm, 0% and 55% porosity: HOYA Technosurgical Inc., Japan) were used to evaluate the hydrophilicity of the HA surface. Four disks of each porosity were treated with UV irradiation for 15 min using a photo device (TheraBeam Affinity; Ushio Inc., Japan) (Fig. 1). The light source mounted in the TheraBeam Affinity is a low-pressure mercury (Hg) lamp, which emits 185-nm and 254-nm UV light. Four disks that did not undergo UV irradiation were used as a control.

To examine the change in hydrophilicity on the disk surface after UV irradiation, the wettability was evaluated by measuring the contact angle of a water drop [3]. For this test, 10 μL of water was dropped onto the disk surface before UV irradiation, and at 0 min, 1 h, 3 h, 6 h, 2 weeks, and 4 weeks after UV irradiation. Moving images were taken at a rate of 240 frames per second (fps) using a high-speed camera (Casio EXILIM EX-ZR 1000, Casio Computer Co., LTD., Japan). Using the still picture immediately after dropping, the angle of the water droplet with respect to the disk surface was measured by the *θ*/2 method [12]. After the water drop landed on the implant surface, the height (a) and contact diameter (b) of the drop were calculated by image analysis software (ImageJ® v.1.48, National Institutes of Health, USA). Using these measurements, the contact angle (*θ*) was calculated with the following formula: *θ* = 2tan − 1 (2a/b). In this analysis, a low contact angle indicates surface hydrophilicity.

### Osteoconduction at hydroxyapatite

Twenty HA cylinders (diameter 2.5 mm, length 8 mm, 55% porosity: HOYA Technosurgical Inc., Japan) were used for the in vivo study. Half of the cylinders were treated with UV irradiation for 15 min as described above. The remaining 10 cylinders were used without UV treatment as a control.

Ten 8-week-old male Sprague–Dawley rats were used for the animal experiments. The rats were anaesthetised with 1 to 2% isoflurane. Both hind limbs were shaved, and the skin and fascia layers were opened separately. The flat aspect of each distal femur was exposed and used for implantation. The right and left distal femurs were drilled using a 3-mm-diameter drill. UV-irradiated HA implants were inserted into the right femur holes, and HA implants without UV irradiation were inserted into the left ones. After implant placement, the skin and fascia were closed. Two or 4 weeks after the surgery, the rats were killed by intraperitoneal injection of pentobarbital, and the femurs were harvested. Five specimens were included in each group.

The specimens were fixed in 10% buffered formalin and analysed using microcomputed tomography (Scan Xmate-L090, Comscantecno Co., Ltd., Japan). The imaging conditions were as follows: voltage, 80 kV; current, 100 μA; magnification, 4.942 times; resolution, 20.234 μm/pixel; and slice thickness, 20.234 μm. Three-dimensional bone morphometric analysis was performed using the software (TRI/3D-BON, RATOC system engineering Co., Ltd., Japan). The mineralised bone volume (BV) ratio and tissue volume (TV) within 100 μm from the implant surface were evaluated. The BV/TV ratio (also called the bone volume fraction), which is an important parameter for evaluating the microstructure of bone, was calculated as the bone volume (%) in this area.

After microcomputed tomography, the specimens were embedded in methyl methacrylate without decalcification. The embedded specimens were then cut perpendicular to the long axis of the implant using a microtome. Each section was stained with Villanueva–Goldner to evaluate the bone area, which was stained green and observed by light microscopy (BZ-X700, Keyence Corp., Japan). To evaluate bone formation around the HA, the bone area (green) for each group was measured in ring-shaped regions 100 μm outside and 100 μm inside the HA surface by digital image analysis software (Image J® v.1.48). The bone area ratio was calculated as the bone area divided by the total measured area (the HA area was subtracted), multiplied by 100 (%).

### Statistical analysis

Two-way analysis of variance with Tukey’s post hoc test was performed to determine differences in the water-drop contact angle. The Wilcoxon signed-rank test was performed to determine differences in the bone volume and bone area ratios between the UV(−) group and UV(+) group at 2 weeks or 4 weeks, respectively. The Mann-Whitney *U* test was also performed to determine differences in the bone volume and bone area ratios at between 2 and 4 weeks within each group. Statistical analyses were performed using SPSS (v 22.0; IBM), and *p* values < 0.05 were considered significant.

## Results

### Hydrophilicity of the HA surface

#### Contact angle analysis

**Fig. 2:**
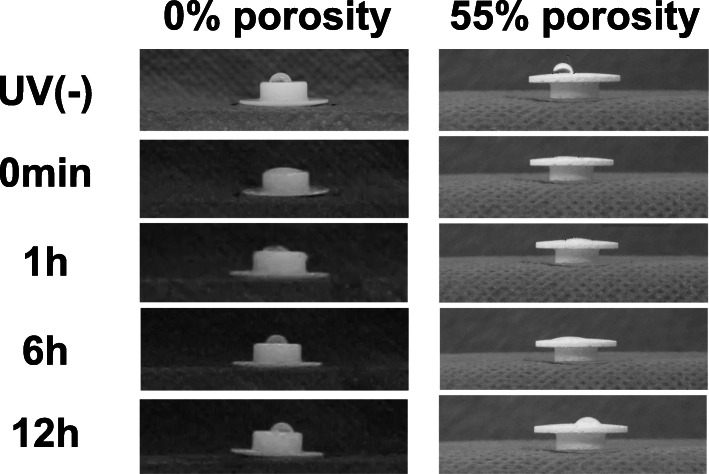
Increased hydrophilicity of the HA surface after UV irradiation as evaluated by measurement of the water-drop contact angle. Photographs show a drop of water deposited onto 0% and 55% porosity HA disks

**Fig. 3:**
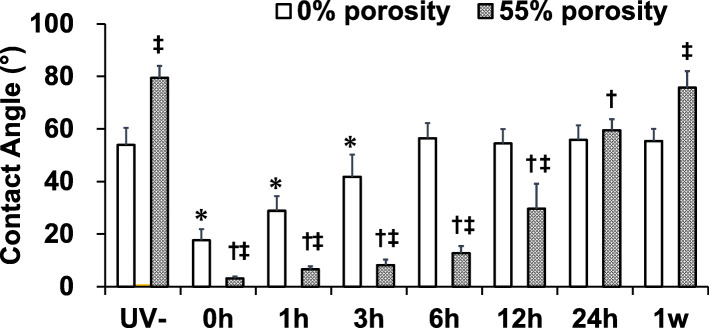
The water-drop contact angle on the HA disk surface indicated hydrophobicity before UV irradiation and hydrophilicity after UV irradiation. The contact angle of both surfaces showed a time-dependent reduction in hydrophilicity. *p < 0.05, *0% porosity UV(−) vs 0 min, 1 h, and 3 h after UV irradiation; ^†^*p* < 0.05, ^†^55% porosity UV(−) vs 0 min and 1, 3, 6, 12, and 24 h after UV irradiation; ^‡^*p* < 0.05, ^‡^0% porosity vs 55% porosity at 0 min; 1, 3, 6, and 12 h; and 1 week after UV irradiation (*n* = 4)

The water-drop contact angle indicated that both 0% porosity HA and 55% porosity HA had a hydrophobic surface before UV irradiation, with a mean contact angle of 53.9° and 79.5°, respectively. After UV irradiation, both surfaces became hydrophilic, with contact angles of 17.7° and 3.2°, respectively (Fig. 2). Over time, the contact angle on both surfaces increased, and the hydrophilicity decreased. In the 0% porosity HA group, there were significant differences between the UV(−) and UV(+) groups immediately, and at 1 and 3 h after irradiation. In the 55% porosity HA group, there were significant differences between the UV(−) and UV(+) groups immediately, and at 1, 3, 6, 12, and 24 h after irradiation (Fig. 3).

### Bone formation around HA samples

#### Bone volume

**Fig. 4:**
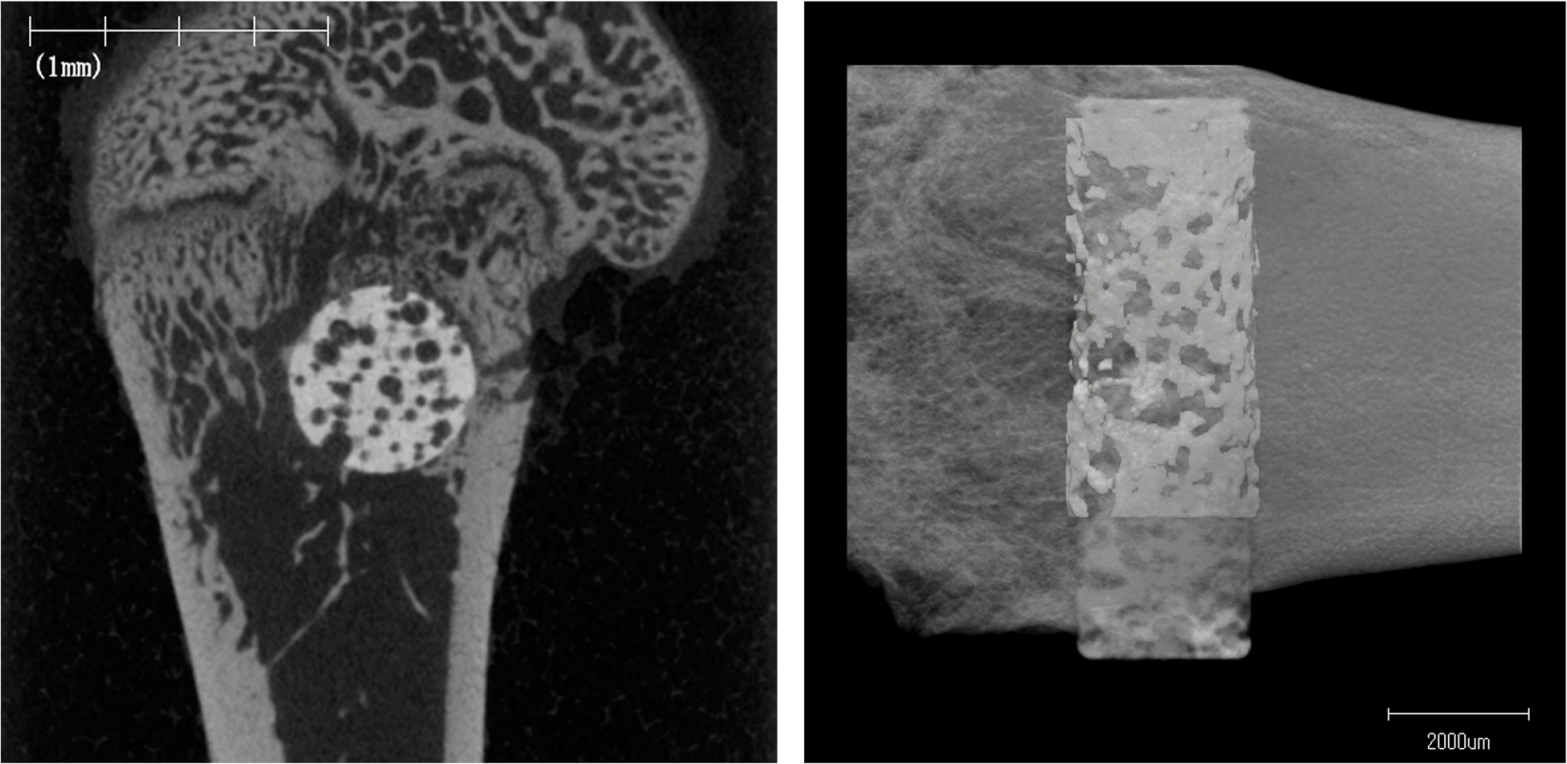
Microcomputed tomography. The right image shows a representative cross-sectional micro CT slice in the perpendicular to the longitudinal axis of the implant. The left image shows a representative three-dimensional computed tomography image around the implant

**Fig. 5:**
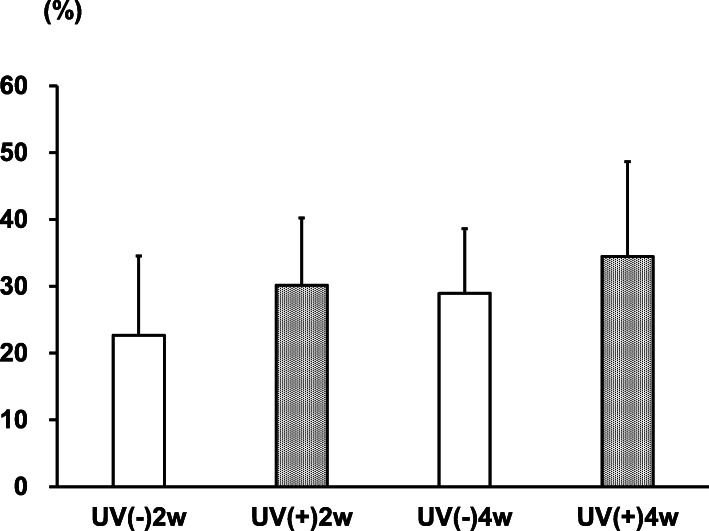
The mean bone volume in the UV-irradiated HA group was higher than that of the non-irradiated group, but the difference was not statistically significant

In the UV(−) group, the mean bone volume was 22.7 ± 11.8% at 2 weeks and 28.9 ± 9.7% at 4 weeks. In the UV(+) group, the mean bone volume was 30.2 ± 10.1% at 2 weeks and 34.5 ± 14.2% at 4 weeks. There was no significant difference between the UV(−) and UV(+) groups at 2 or 4 weeks (*p* = 0.080, *p* = 0.345) (Figs. 4 and 5). An increase in bone volume was observed over time in each group; however, it was also not statistically significant difference (*p* = 0.421, *p* = 0.754).

#### Histological analysis

**Fig. 6:**
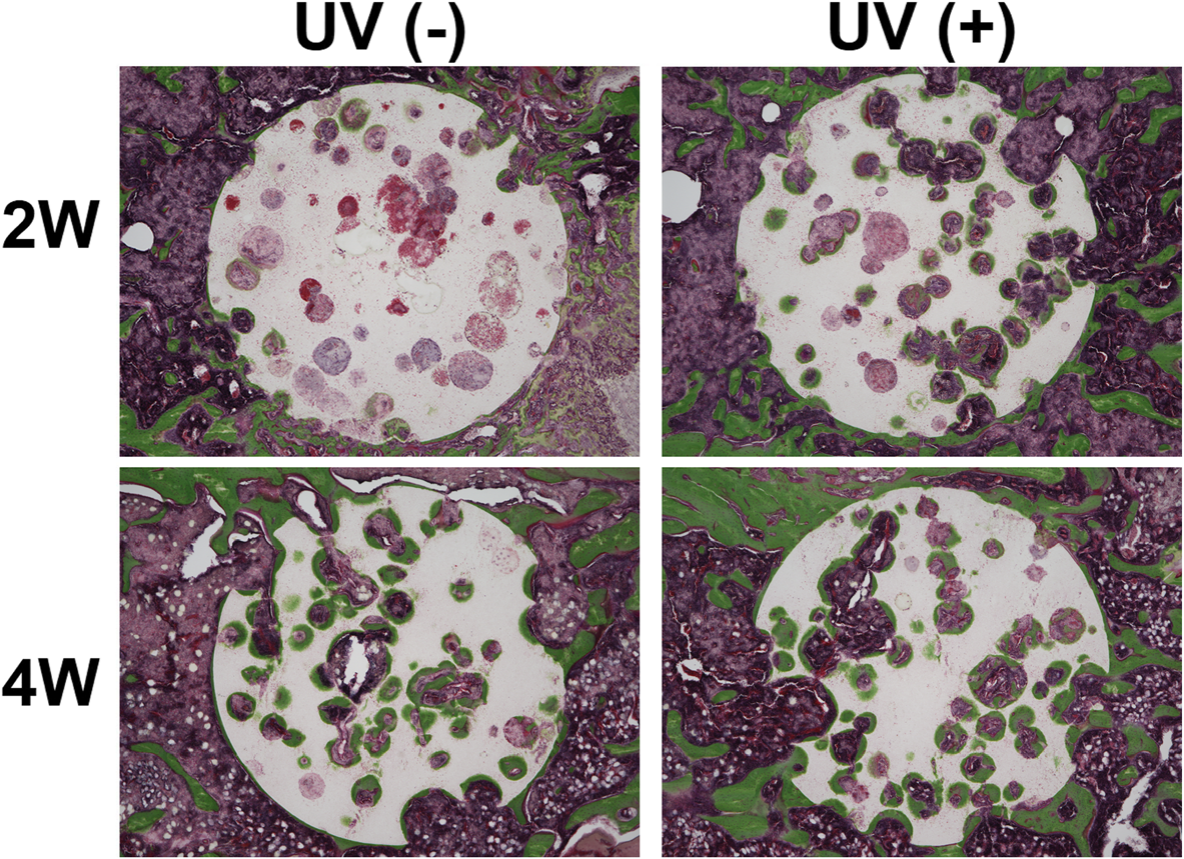
Light microscopy images of Villanueva-Goldner staining 2 and 4 weeks after the implantation of HA into rats

**Fig. 7:**
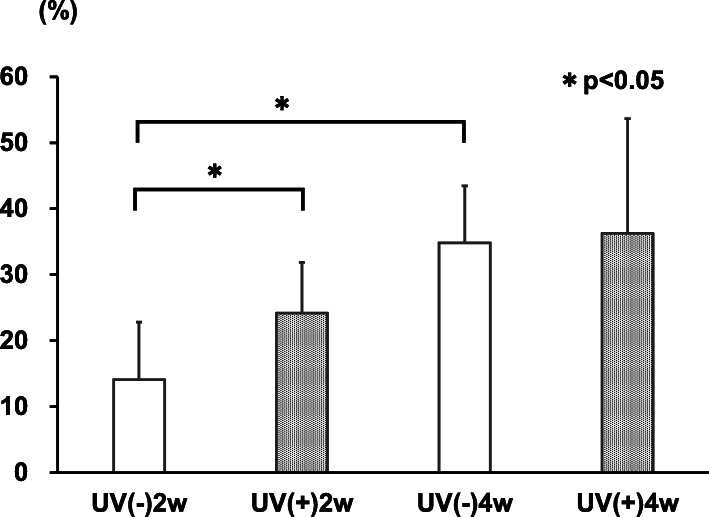
The mean bone area ratio in the UV-irradiated HA group was statistically significantly higher than that of the non-irradiated group at 2 weeks; however, there was no significant difference at 4 weeks

In the UV(−) group, the mean bone area ratio was 14.1 ± 7.5% at 2 weeks and 34.8 ± 8.6% at 4 weeks. In the UV(+) group, the mean bone area ratio was 24.2 ± 6.7% at 2 weeks and 36.3 ± 17.4% at 4 weeks. There was a significant difference between UV(−) and UV(+) groups at 2 weeks (*p* = 0.043), but not at 4 weeks (*p* = 0.893) (Figs. 6 and 7). A statistically significant increase in bone area ratio was observed over time in the UV(−) group (*p* = 0.009); however, there was no statistically significant increase in the UV(+) group (*p* = 0.175).

## Discussion

This study demonstrated that photofunctionalisation changed the HA surface from hydrophobic to hydrophilic regardless of the porosity and that the hydrophilicity was maintained for at least a few hours. The bone area in the early phase (at 2 weeks) after implantation was significantly higher in the UV(+) than in the UV(−) group.

There are several reports on the effect of photofunctionalised Ti, which is a medical material. Photofunctionalised Ti implants are reported to increase the bone-implant contact ratio 2.5 times after 2 weeks, and 1.9 times after 4 weeks compared to Ti without photofunctionalisation [1]. In our previous study, Yamauchi et al. reported that UV-treated Ti and Ti6Al4V demonstrated significant differences in chemical properties and were more wettable than untreated implants [3]. Furthermore, we showed that antimicrobial activity was induced on Ti and Ti6Al4V for 7 days after UV irradiation [8]. UV irradiation removes carbon deposition from the Ti surface and exposes Ti4+ sites. It enhances the bioactivity of the surface by increasing its wettability and hydrophilicity. These changes enhance the initial attachment, proliferation, and differentiation of osteoblasts [1, 13].

This study demonstrated that the bone area around HA was greater in the UV-irradiated group than that in the control group at 2 weeks postoperatively. There are a few reports on the effects of ultraviolet irradiation on HA. Nishikawa reported that O_2_^−^ is generated by electron transfer to O_2_ after irradiating HA and that OH is produced by the reaction of O_2_^−^ and H_2_O [8]. It was proposed that the radical action in atmospheric air causes the decomposition of organic pollutants similar to the TiO_2_ photocatalyst [8]. Wakamura et al. investigated the effect of irradiating Ti-modified HA on the killing of colon bacillus [9]. They showed that Ti-modified HA exhibits a higher bactericidal effect than TiO_2_ and that Ti-modified HA has both an absorption affinity for and a photocatalytic activity against microorganisms. They proposed that irradiation forms positive holes, which interact with absorbed H_2_O to yield hydroxyl radicals with a strong oxidation ability, which can decompose various organic materials as a bactericidal effect. Tanaka et al. analysed the decomposition of dimethyl sulphide on HA after UV irradiation using infrared spectroscopy and showed that the area intensity of CH bands due to dimethyl sulphide gradually decreases and that of surface P-OH bands increases after irradiation [10]. These findings meant that surface P-OH- radicals were formed, and dimethyl sulphides were decomposed by UV irradiation. Taken together, these previous reports showed that UV irradiation physicochemically alters the HA surface. Hydroxyl radicals on the surface of irradiated HA can increase its wettability and hydrophilicity, similar to the effect of photofunctionalised Ti. In an in vitro study using HA/PLLA, UV treatment improved the surface hydrophilicity without changing the mechanical strength, cell adhesion to UV-treated HA/PLLA was significantly improved, and cell differentiation was also significantly increased [11]. The cell-adhesion ability may increase due to the change in wettability and hydrophilicity, leading to the improved osteoconductive ability of HA in the early phase.

HA is widely used as a coating for uncemented total hip arthroplasty components. HA is an osteoconductive coating that has been shown to enhance implant fixation and accelerate bone growth [13, 14]. Autopsy retrievals showed the presence of extensive circumferential bone apposition on a HA-coated Ti femoral stem [15] and more bone ingrowth around femoral stems with HA coating than around those without HA [16]. The long-term follow-up of HA-coated stems has shown excellent clinical and radiographic outcomes [17–19]. A meta-analysis showed that HA-coated stems had better clinical scores and implant survival than porous-coated stems [20]. On the other hand, Schewelov et al. reported that some stem subsidence of fully HA-coated stems occurred [21]. In addition, in patients with a femoral neck fracture, 31 of 38 HA-coated stems migrated distally (mean value 2.7 mm) during the first 3 months [22]. Our findings suggest that the UV irradiation of HA-coated stems might induce earlier biological fixation and prevent stem subsidence.

Iguchi et al. reported that high porosity HA-spacer-augmented laminoplasty produced good bonding-related results, as evaluated by computerised tomography [23]. However, they also reported that 4.4% of the spacers broke. Ono et al. reported two cases of dural damage from the dislocation of HA spacers due to absorption of the tip of the spinous process after cervical laminoplasty. Both patients underwent removal of the HA spacer and attained good neurological recovery [6]. Therefore, early union of the bone and HA spacer may enable early range of motion of the neck after cervical laminoplasty.

This study had some limitations. First, the biomechanical strength of the bone-HA integration was not tested. Second, the relationship between hydrophilicity and osteoconductivity after UV irradiation was not analysed. In addition, there are no data relative to cell adhesion or proliferation on HA after UV irradiation. Third, only five specimens were included in each group, making it difficult to come to a statistical conclusion. Post hoc power analysis indicated that five specimens provided a power of 0.6 to detect the difference of bone area between UV(−) and UV(+) groups (effect size = 1.3, *α* = 0.05). Finally, the surfaces of the UV-irradiated and non-irradiated HA were not evaluated by electron microscopy. Further studies are needed to confirm the biomechanical strength of the UV-irradiated HA and the mechanism that promotes osseointegration in the early healing stage.

## Conclusion

This study revealed that the surface of UV-irradiated HA disks were hydrophilic after UV irradiation, in contrast to the non-irradiated HA disks. Photofunctionalisation induced an accelerated increase in the bone area ratio in the early healing stage in rats. This technology could be applied to surgical cases requiring early fusion of the bone with HA.

## Data Availability

The datasets used and analysed during the current study are available from the corresponding author on reasonable request.

## Abbreviations

UV: Ultraviolet
HA: Hydroxyapatite
Ti: Titanium
Ti6Al4V: Titanium alloy
BIC: Bone-implant contact
BV: Bone volume
TV: Tissue volume
